# Inhaled anti-thymic stromal lymphopoietin antibody fragment, ecleralimab, in severe asthma: A placebo-controlled, multicenter, double-blind, randomized, phase 2b clinical trial

**DOI:** 10.1093/ajrccm/aamag123

**Published:** 2026-04-08

**Authors:** Alexander J Mackay, Christian Gessner, Horacio Budani, Steven Weinstein, Yasushi Fukushima, Erika A Zannou-Mantri, Ilias Stavropoulos, James M Felser, Keroles Nakhla, Peter D’Andrea, Xintong Chen, Paul M O’Byrne

**Affiliations:** Novartis Pharma AG, Basel, Switzerland; Department of Clinical Immunology, University Hospital Leipzig, Leipzig, Germany; POIS Leipzig GbR, Leipzig, Germany; Respiratory Area of the Foundation for Clinical Assistance and Research in Prevalent Diseases (FAICEP), Buenos Aires, Argentina; Allergy and Asthma Specialists Medical Group and Research Center, Huntington Beach, CA, United States; Fukuwa Clinic, Tokyo, Japan; Novartis Pharmaceuticals Corporation, East Hanover, NJ, United States; Novartis Ireland Limited, Dublin, Ireland; Novartis Pharmaceuticals Corporation, East Hanover, NJ, United States; Novartis Pharmaceuticals Corporation, East Hanover, NJ, United States; Novartis Pharmaceuticals Corporation, East Hanover, NJ, United States; Novartis Pharmaceuticals Corporation, East Hanover, NJ, United States; Department of Medicine, McMaster University, Hamilton, ON, Canada

**Keywords:** Human TSLP, Asthma, Lung function

Asthma is characterized by reversible bronchoconstriction and exaggerated airway responses to bronchoconstrictor stimuli. Despite advances, there is still an unmet treatment need.^1^ The epithelial cell–derived cytokine thymic stromal lymphopoietin (TSLP) is a key regulator of T-helper type 2 inflammation.^2^ Tezepelumab, an anti-TSLP monoclonal antibody, has shown benefit in patients with severe uncontrolled asthma when administered by injection.^3^ Inhaled anti-TSLP therapies might yield greater clinical benefit by delivering more rapid and/or superior local lung targeting and overcome potential patient hesitancy caused by needle phobia.

Ecleralimab (CSJ117) is a potent neutralizing antibody fragment (fragment antigen binding) directed against human TSLP. An allergen bronchoprovocation proof-of-concept study showed that ecleralimab, delivered using Breezhaler^®^, significantly attenuated allergen-induced bronchoconstriction and airway inflammation.^4^

To evaluate the safety and efficacy of ecleralimab, we conducted a placebo-controlled, multinational, multicenter, double-blind, randomized, parallel-arm, phase 2b study. Five doses of ecleralimab inhaled once daily, when added to standard-of-care asthma therapy, were assessed in adult subjects (aged 18 years and older and younger than 75 years) with inadequately controlled asthma, receiving medium- to high-dose inhaled corticosteroids plus long-acting beta_2_ agonist, with up to 2 additional asthma controller medications (ClinicalTrials.gov: NCT04410523). Randomly assigned patients had an Asthma Control Questionnaire-5 (ACQ-5) score of  ≥ 1.5 and prebronchodilator forced expiratory volume in 1 second (FEV_1_) of  ≥ 40% and  ≤ 85%.

The study involved a 2-week screening, followed by a 4-week single-blinded placebo run-in, a 12-week double-blinded treatment, concluding with an 8-12 week follow-up. The primary endpoint was the average change from baseline (CFB) in predose, prebronchodilator FEV_1_ at week 8 and week 12. A key secondary endpoint included assessment of the CFB in fractional exhaled nitric oxide (FeNO). The full analysis set (patients who received at least 1 dose of treatment) was used. Multiple comparison procedure, modeling methodology was used for the primary endpoint.^5^^,^^6^ Adjusted mean responses were estimated using a mixed model for repeated measures for both endpoints. Antidrug antibody (ADA) titers and adverse events (AEs) were also measured.

Initially, 625 patients were to be randomly assigned; however, the study was terminated early due to strategic business reasons by the sponsor with 335 patients randomly assigned. No adverse safety signals impacted the decision to terminate the study, and no efficacy data were evaluated before termination.^7^ Patients were randomly assigned to receive either ecleralimab 0.5, 1, 2, 4, or 8 mg or placebo. Overall, 304 patients completed the study treatment, and 31 prematurely discontinued. Patient demographics were well balanced between the groups (Table 1). Treatment compliance, determined by the investigational staff, was very good (80%-100%).

**Table 1:** Patient demographics and baseline characteristics (randomized set).

Characteristics	Ecleralimab 8 mg	Ecleralimab 4 mg	Ecleralimab 2 mg	Ecleralimab 1 mg	Ecleralimab 0.5 mg	Placebo
	**(*n*** = **74)**	**(*n*** = **76)**	***(n*** * = * **37)**	**(*n*** = **37)**	**(*n*** = **36)**	**(*n*** = **75)**
**Age, mean ± SD, y**	52.7 ± 12.22	50.3 ± 12.80	51.4 ± 13.31	51.2 ± 12.09	49.8 ± 12.89	51.5 ± 13.46
**Female, No. (%)**	48 (64.9)	43 (56.6)	21 (56.8)	25 (67.6)	27 (75.0)	45 (60.0)
**Body mass index, mean ± SD, kg/m^2^**	27.95 ± 5.67	28.41 ± 6.74	27.63 ± 5.23	29.29 ± 7.81	28.48 ± 6.35	28.20 ± 6.61
**Duration of asthma, mean ± SD, y**	22.49 ± 14.62	24.70 ± 15.08	24.46 ± 13.19	23.91 ± 14.30	25.23 ± 15.54	24.16 ± 14.53
**ACQ-5 score, mean ± SD**	2.33 ± 0.53	2.42 ± 0.60	2.20 ± 0.49	2.34 ± 0.60	2.40 ± 0.55	2.36 ± 0.62
**FeNO, mean ± SD, ppb**	28.3 ± 20.51	26.8 ± 22.54	36.5 ± 25.99	26.7 ± 20.18	22.1 ± 14.77	26.7 ± 21.22
**FEV_1_, mean ± SD, L^a^**	1.96 ± 0.49	2.03 ± 0.52	2.04 ± 0.47	2.03 ± 0.56	2.00 ± 0.65	1.99 ± 0.58
**Percent predicted FEV_1_, mean ± SD, prebronchodilator**	65.68 ± 11.37	64.19 ± 11.45	66.15 ± 9.61	64.76 ± 9.60	65.19 ± 12.77	64.45 ± 11.04
**No. of exacerbations in the prior year, No. (%)**						
** 0**	1 (1.4)	0	1 (2.7)	0	1 (2.8)	1 (1.3)
** 1**	64 (86.5)	66 (86.8)	29 (78.4)	28 (75.7)	28 (77.8)	60 (80.0)
** 2**	8 (10.8)	8 (10.5)	7 (18.9)	9 (24.3)	7 (19.4)	13 (17.3)
** ≥3**	1 (1.4)	2 (2.6)	0	0	0	1 (1.3)
**Blood eosinophil count, mean ± SD, cells/μL**	306.0 ± 190.94	312.5 ± 254.20	406.8 ± 293.25	333.2 ± 262.70	361.1 ± 293.87	316.3 ± 187.90
**Serum IgE level, mean ± SD, μg/L**	479.14 ± 691.29	690.99 ± 1630.92	514.11 ± 1005.51	285.70 ± 342.46	371.34 ± 453.15	593.90 ± 941.02

a Baseline is defined as the average of 2 h 15 min predose and 2 h 45 min predose assessments at the end of the run-in visit. Abbreviations: ACQ, Asthma Control Questionnaire; FeNO, fractional exhaled nitric oxide; FEV_1_, forced expiratory volume in 1 second; IgE, immunoglobulin E; n, number of participants; ppb, parts per billion; SD, standard deviation.

Ten patients reported on-treatment AEs suspected of being related to the study treatment (ecleralimab 0.5 mg: 0% [0/36]; 1 mg: 2.7% [1/37]; 2 mg: 2.7% [1/37]; 4 mg: 2.6% [2/76]; 8 mg: 5.4% [4/74]; and placebo: 2.7% [2/75]); none were serious. Most common AEs were cough and dry mouth, occurring in 2 subjects treated with ecleralimab 8 mg. No deaths were reported in this study. Three serious on-treatment AEs occurred in 2 subjects (1 ecleralimab 0.5 mg and 1 placebo), but none were considered to be related to the study treatment by the investigator.

The study did not demonstrate a dose–response relationship for FEV_1_ or FeNO; therefore, the efficacy of the treatment was not proven. In exploratory analyses, positive signals were seen at low ecleralimab doses. The greatest numerical improvement, compared with placebo, in FEV_1_ was seen at 0.5 mg (treatment difference [Δ], 0.122 (L); [95% Confidence Interval (CI), 0.016 to 0.229]; Figure 1A) and in FeNO at 1 mg ([Δ] −3.998 (ppb); [95% CI, −8.691 to 0.695]; Figure 1B), in contrast to the lack of improvement seen with the 4- or 8-mg doses. No clinically meaningful improvement was observed in ACQ-5 scores or rescue inhaler usage.

**Figure 1:**
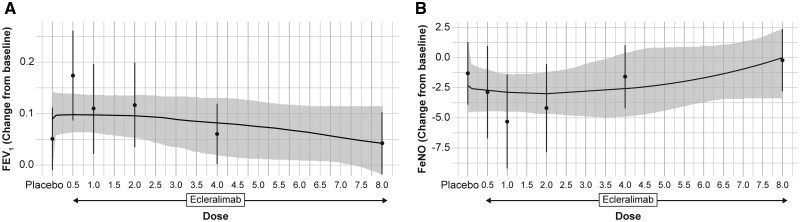
(A) Mean change from baseline in predose FEV_1_ (L) averaged between weeks 8 and 12 (full analysis set). (B) Mean change from baseline in FeNO (ppb) averaged between weeks 8 and 12 (full analysis set). The line represents the median of the bootstrapped dose-response curve. The band represents the range between 2.5% quantile and 97.5% quantile of the bootstrapped dose-response curve. The dots and error bars are the LS mean estimates and 95% confidence intervals for the mean change from baseline in (A) predose FEV_1_ (B) predose FeNO based on the MMRM model. Dose units (mg). Abbreviations: FEV_1_, forced expiratory volume in 1 second; L, liter; FeNO, fractioned exhaled nitric oxide; LS, least squares; MMRM, mixed models for repeated measures; ppb, parts per billion.

Earlier studies showed clinical benefit of anti-TSLP therapy when administered systemically.^3^ The results of the ecleralimab bronchoprovocation study were also encouraging.^4^ Unfortunately, partly due to the early termination of the study, it is not possible to define the reason(s) for the results observed. The current analysis was underpowered, and full recruitment of the study may have yielded better efficacy results.

The immunogenicity of ecleralimab may have a negative impact on its efficacy. The systemic levels of ecleralimab were very low, and ADA levels increased with increase in dose over time (observed data). The ADA assay was based on a bridging electrochemiluminescence immunoassay method using a MesoScale Discovery plate reader system to detect anti-CSJ117 antibodies in human serum. Antidrug antibodies were detected in 67%-86% of patients by week 12, persisting until week 24. However, it is not possible to conclude the ADAs were neutralizing and were the driver for the poor efficacy observed. Positive ADA results were also seen in 16% of placebo-administered patients and 8%-17% of predose ecleralimab patients.

The lung plays a critical role in host immunity, striking a balance between tolerance to particulate matter, while concurrently providing protection from respiratory pathogens.^8^ It is possible that ADAs are a more likely consequence of a lung-targeted delivery approach. Antidrug antibodies have the potential to inactivate drugs, leading to loss of efficacy.^9^ A study examining aerosol administration of anti-IgE monoclonal antibody, omalizumab, did not show inhibition of airway responses to inhaled allergen in asthmatic subjects, in contrast to positive results seen using the parenteral approach. The authors hypothesized the lack of efficacy may have been due to the development of neutralizing antibodies, because aerosol delivery may be more immunogenic than parenteral approaches.^10^ Future studies will need to clarify whether failure of ecleralimab treatment is specific to this molecule and/or a study limitation. If the failure was driven by the negative impact of immunogenicity, then similar inhaled biologic approaches may need to overcome this limitation to be successful.

In conclusion, ecleralimab was safe and well tolerated but did not demonstrate a dose-response relationship for the efficacy endpoints; therefore, efficacy was not proven. High levels of ADAs may have contributed to the negative findings. However, due to early termination and incomplete enrollment, it is difficult to draw definitive conclusions.

## Supplementary Material

aamag123_Supplementary_Data

## Data Availability

Novartis is committed to sharing with qualified external researchers, access to patient-level data, and supporting clinical documents from eligible studies. These requests are reviewed and approved by an independent review panel on the basis of scientific merit. All data provided are anonymized to respect the privacy of subjects who have participated in the trial in line with applicable laws and regulations. Result summaries have been posted on the Novartis clinical trial database and other online public databases. More information on Novartis’ position on access to clinical trial results and patient-level data is available at https://www.novartis.com/our-science/clinical-trials/clinical-trial-information-disclosure.
